# GIANT: A Cytoscape Plugin for Modular Networks

**DOI:** 10.1371/journal.pone.0105001

**Published:** 2014-10-02

**Authors:** Fabio Cumbo, Paola Paci, Daniele Santoni, Luisa Di Paola, Alessandro Giuliani

**Affiliations:** 1 Institute for System Analysis and Computer Science “Antonio Ruberti”, National Research Council, Rome, Italy; 2 SysBio Centre for Systems Biology, Milan and Rome, Italy; 3 Faculty of Engineering, Università CAMPUS BioMedico, Roma, Italy; 4 Environment and Health Department, Istituto Superiore di Sanità, Roma, Italy; Universitat Pompeu Fabra, Barcelona Research Park of Biomedicine (PRBB), Spain

## Abstract

Network analysis provides deep insight into real complex systems. Revealing the link between topological and functional role of network elements can be crucial to understand the mechanisms underlying the system. Here we propose a Cytoscape plugin (GIANT) to perform network clustering and characterize nodes at the light of a modified Guimerà-Amaral cartography. This approach results into a vivid picture of the a topological/functional relationship at both local and global level. The plugin has been already approved and uploaded on the Cytoscape APP store.

## Introduction

The network paradigm helps modeling the multiscale character of biological systems: “networks” is the generic name for graphs, which represent a set of nodes linked by edges. Complex systems are, thus, easily represented by graphs, whose nodes are the system elements and edges represent the relation between them.

The network structure allows for a natural combination of different scales: each node inherits its role in the system by its location in the network (top-down causation), while the global properties of the whole network depend upon the edges (bottom-up causation).

Biological networks (e.g., protein-protein interaction networks, protein contact maps, gene expression networks, ) very often display a scale-free architecture lying halfway between random networks, whose wiring is assigned according to a Gaussian distribution of link probability, and regular networks, whose nodes all show the same degree (number of edges pertaining to a single node). One of the most challenging tasks for biological scale-free networks analysis is to assign a functional role to each node depending on its location in the network.

In their innovative work, Han *et al.* [1] estimated the dynamics of hubs (high-degree nodes) from the analysis of messenger RNA expression profiles. The authors examined how much hubs in the yeast interactome are co-expressed with their interaction partners, computing the average Pearson correlation coefficient (APCC) between the hub mRNA expression and its nearest neighbors. They found APCC distribution follows a bimodal distribution singling out two distinct hub populations: they called “party hubs” those nodes that are highly correlated in expression with their partners (high APCC) and “date hubs” those showing more limited co-expression with their own partners (lower APCC). This distinction matches with permanent (party hubs) and transient interactions (date hubs) [1]. Eventually, the authors showed that a link exists between this hub classification and the network tolerance against node breakdown: scale-free networks are particularly resilient to random node removal (failure), albeit extremely sensitive to the targeted removal of hubs (attack) [2], [3].

The work by Han et al. [1] is just one out of many applications of network approach in the biology and biotechnology realm (see [4] for a comprehensive review). The by far most part of the network applications deals with the mesoscopic properties of the graphs representing a link between structural and functional properties of systems.

In their seminal work [5], Guimerà and Amaral developed a methodology for the multiscale network analysis passing by the network module identification (network clustering): they classified nodes according to their inter and intra-module connectivity, by identifying two descriptors, the participation coefficient *P* and the within-module z-score *z*, for the inter and intra-module connectivity, respectively. This method has been largely applied in many different fields, from metabolic networks [5] to brain functionality [6], [7], passing by non biological application [8]. The analysis of 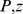 space shows peculiar features when derived for protein contact networks [9]–[12], providing a meaningful functional characterization of local and global network properties.

Here we propose a Cytoscape plugin, GIANT (GuImerà Amaral NeTwork) implementing our modified interpretation of the Guimerà and Amaral cartography. This plugin identifies modules in a network by three different clustering methods: spectral, k-means and MCL (Markov CLuster) algorithm. The proposed approach fits with any clustering algorithm, such as those implemented in clusterMaker [13]. The output is the network cartography in the 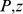 plane, highlighting nodes role according to our modified Guimerà and Amaral classification.

We show the application to two case studies of biological relevance: the protein contact network of hemoglobin and the co-expression network of *Vitis Vinifera*. The color map superimposed to the Cytoscape network view shows a clear relation between the nodes role and their 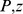 description.

## Materials and Methods

**Figure 1:**
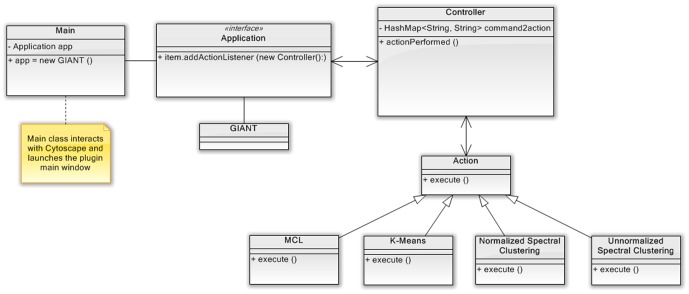
GIANT classes flow chart. Action classes are shown for the clustering algorithms implemented in the plugin: spectral clustering, MCL and k-means. Spectral clustering can be run in two mode, normalised and unnormalized. Each clustering action class starts the related algorithm in a new thread to maximize the performance.?

The plugin runs through the Cytoscape software [14] and allows the developers to use it as an independent Java library and to implement custom software. The installation of GIANT is possible directly via the Cytoscape plugin manager (menu Plugin 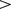 Manage Plugins, section Clustering, selecting the latest version of GIANT); alternatively, the plugin, along with the source code and video tutorials, is directly accessible for download from the GIANT official website http://www.iasi.cnr.it/~dsantoni/GIANT/giant.html. GIANT has been developed following the classical MVC (Model-View-Controller) pattern. Figure 1 shows the GIANT *UML* class diagram and the process execution when clustering analysis is launched. The *Main* class provides the integration with the Cytoscape environment and drives the plugin user interface (*GUI*) (GIANT class implements the generic interface *Application*). Each *GUI* event, provided by the plugin, connects to a controller that maps it to a specific action. All classes that represent an action extend the abstract class *Action* (and all its methods). Figure 1 reports the action classes, corresponding to the clustering algorithms in the plugin. Each clustering action class starts the related algorithm. The clustering algorithms are developed in the *JavaML* library included in GIANT: the library is written in Java and is available from www.java-ml.sourceforge.net/under the GNU GPL license. The library implements a collection of machine learning and data mining algorithms, readily usable and easily extensible *API* for both software developers and research scientists. The algorithms strictly follow their respective interfaces, that are user-friendly and simple.

GIANT has been accepted by the Cytoscape community and is presently available for the download on the official Cytoscape APP Store http://apps.cytoscape.org/apps/giant. The plugin was successfully tested on the 2.8 and 3 releases of Cytoscape and it was tested on Windows, Linux and OS X operating systems ( in 5 or higher version of the Java Runtime Environment).

### 2.1 Clustering algorithms

The plugin implements three clustering algorithms: spectral Meila-Shi [15], MCL [16] and k-means [17]. It is worth noting the cartography is totally independent from clustering algorithm: the user can upload an already determined partition.

#### 2.1.1 Spectral clustering algorithm

The spectral clustering algorithm takes as input the unweighted adjacency matrix of the network and the number *k* of clusters. Graph nodes are partitioned according to the components of the first *k* eigenvectors.

The first step is to compute the Laplacian matrix *L* depending on the unweighted adjacency matrix *A*:
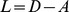



*D* is the diagonal degree matrix, whose generic element 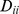 is the i-th node degree. The algorithm applies either to the unnormalized or the normalized Laplacian matrix.

Once the eigenvectors 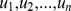 of the Laplacian matrix *L* are computed, a matrix 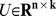 is built, whose 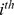 column corresponds to the 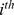 eigenvector. The rows of matrix *U* correspond to nodes, partitioned into clusters according to their coordinates in matrix *U* by means of the k-means algorithm (see next subsection).

#### 2.1.2 k-means algorithm

k-means is an unsupervised learning and partitioning clustering algorithm. The algorithm aims at minimizing the objective function Sum of the Squared Error (SSE), i.e. the sum of the squared distances of each node from the closest cluster centroid:
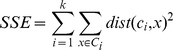
where *k* is the number of the clusters, 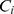 is the 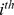 cluster, *x* is an object (*i.e.* a point in the 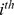 cluster), 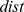 is the standard Euclidean (L2) distance between two objects in a Euclidean space and 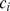 is the centroid of the 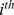 cluster. It is easy to demonstrate the best centroid (minimum cluster's SSE) is the cluster center of mass:



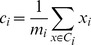




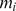
 being the number of nodes in the 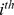 cluster. The abscissa of the elbow in the SSE vs. *k* plot represents the optimal number of clusters. In GIANT, k-means acts on distances between nodes adopting as metrics the shortest paths and the Hamming distances computed on the adjacency matrix.

#### 2.1.3 Markov Cluster algorithm

The MCL algorithm is a fast and scalable unsupervised cluster algorithm for networks based on simulation of (stochastic) flow in graphs. The algorithm simulates a flow on the graph to compute next powers of the associated adjacency matrix. At each iteration, an inflation step is applied to enhance the contrast between regions of strong and weak flow in the graph. The process converges towards a partition of the graph, with a set of high-flow regions (the clusters), parted by boundaries with no flow. The value of the inflation parameter strongly influences the number of clusters.

### 2.2 Node classification

Once the modules (clusters) are identified, the intra and inter-module connectivities are represented respectively by:

• the within-module z-score [18]

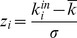
where 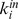 is the the number of links of node *i* with nodes in its own module, 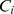, 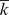 and 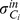 are, respectively, the average value and the standard deviation of the overall degree distribution;

• the participation coefficient (modified with respect to its original definition [18] ) describes the attitude of the node to connect to nodes in modules other than theirs:
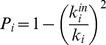




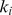
 being the total degree of node *i*.

We adopted a modified version of Guimerà and Amaral participation coefficient *P* [18], that has helped us to identify nodes in the R4 region of the Guimerà-Amaral cartography (work in progress) not detectable by the original algorithm. R4 region collects nodes with fewer than 35% of their links within their own module, *i*.e., with a strong inter-cluster connectivity character (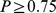).

**Figure 2:**
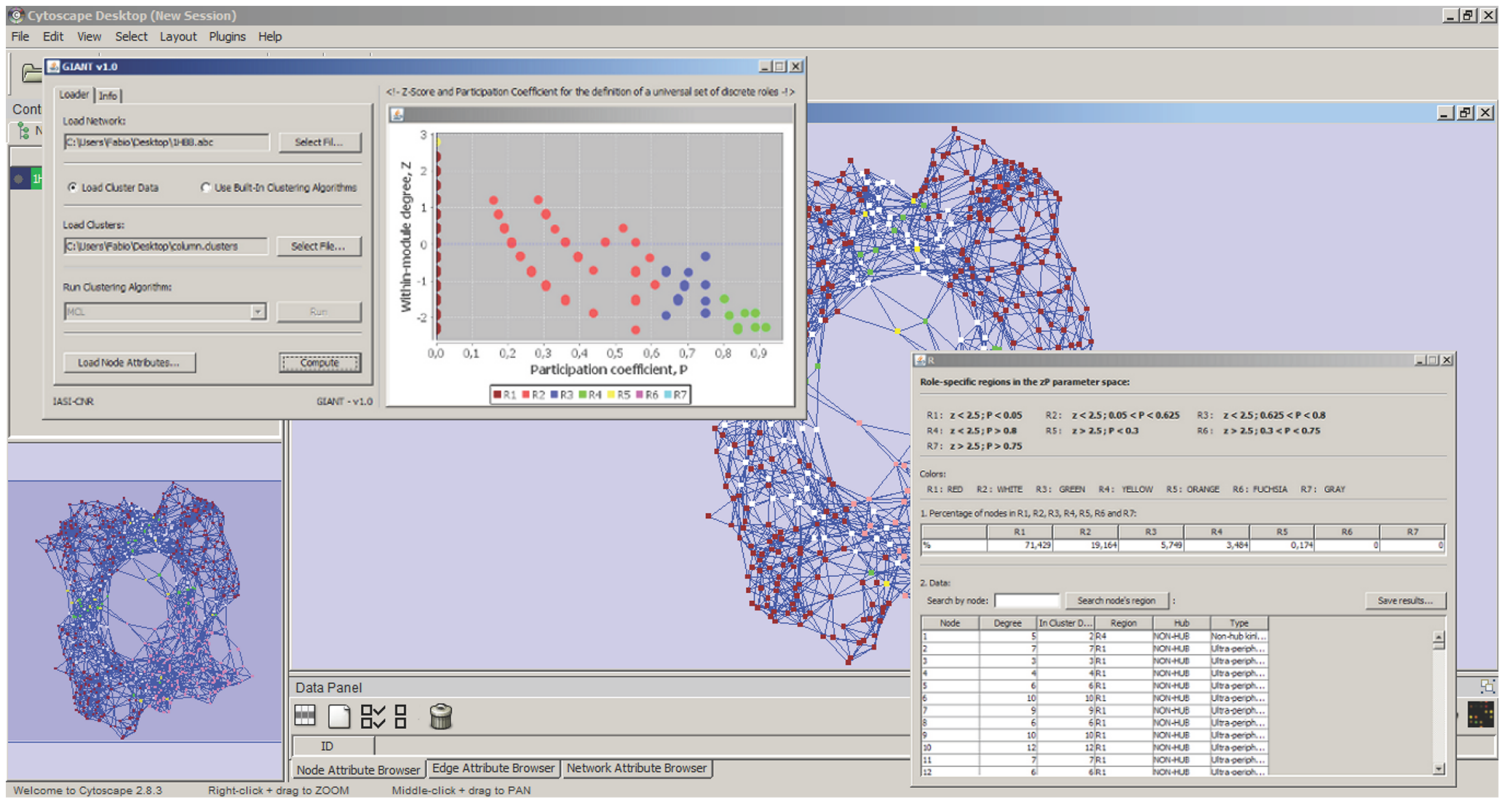
Screenshot of GIANT plugin. GIANT outcome to the hemoglobin (PDB code 1HBB) protein contact network application: the Guimerà-Amaral cartography, the protein contact network and the output table are reported. The nodes color corresponds to their role in the cartography (see Table 1).

The original Guimerà and Amaral definition of *P* [18] approaches to an upper threshold corresponding to node links uniformly distributed among modules (*i*.e., 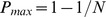, where *N* is the number of modules). Thus, for instance, when we part the hemoglobin contact network into 4 modules, 0.75 is the maximum value for *P*, corresponding to the lower bound of the R4 region. Therefore, if we had used the original definition of *P*, we could not highlight the R4 nodes corresponding to the sub-units contacts, which in turn have a high functional relevance in the protein structure (connector nodes colored in black in Figure 2C).

According to 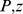 values, the plugin reports the node classification (see Table 1): ‘module hubs’ (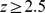, *i.e.* hubs within their own module), ‘module non hubs’ (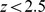, *i.e.* node that are not hubs within their module but that can still have a hub role in the whole network). According to the participation coefficient *P*, the non-hubs nodes can be divided into four regions (R1, R2, R3, R4) and the hubs nodes into three regions (R5, R6, R7).

**Table 1:** Guimerà - Amaral cartography: nodes role.

	Regions	Within-module z-score	Participation coefficient
module non-hubs	R1: Ultra-peripheral node	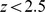	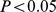
	R2: Peripheral node	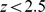	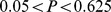
	R3: Non-hub connectors	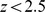	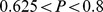
	R4: Non-hub kinless nodes	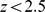	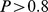
module hubs	R5: Provincial hubs	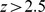	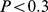
	R6: Connector hubs	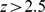	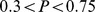
	R7: Kinless hubs	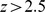	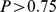

### 2.3 User's Guide

As a first step, the user must upload the network in.abc format: this file format requires two fields separated by a white space on each line. The first and second fields are labels specifying source and destination node, respectively. The file can contain an optional third column representing the nodes interaction. A file containing clusters data can be loaded, if available. The file to upload must be .idx type, containing two columns with the ID of the nodes and the relative cluster indication; alternately, the user can use the built-in clustering algorithms. K-means algorithm is based on two different metrics: shortest paths and Hamming distances, computed on the nodes adjacency vectors. Spectral clustering relies on two running modes: normalized and un-normalized.

Once the network is uploaded, the user must check the *Use Built-in Clustering Algorithm* option and choose the *kMeans* algorithm. In the *KMEANS INITIALIZER* left panel, there are two options: in the *Cluster Validation* section, once defined the minimum *k_min* and the maximum *k_max* number of clusters, within this range the Sum Square Error as a function of *k* (number of clusters) is displayed. The second option is that the user can directly decide the number of clusters *k*. The user can also upload, if any, a file with node attributes as required by Cytoscape environment for network visualization.

Once data are loaded and clusters made available, the Guimerà-Amaral cartography is computed (see previous section). Results are provided as a table containing a summary of 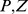 values associated to each node as well as node degree, the region *R* (according to Guimerà-Amaral cartography) and all other features uploaded as node attributes. Practical video tutorials can be downloaded from the web site http://www.iasi.cnr.it/~dsantoni/GIANT/giant.html. GIANT is downloadable directly from Cytoscape APP store http://apps.cytoscape.org/apps/giant or from its official web site: http://www.iasi.cnr.it/~dsantoni/GIANT/giant.html, where a video tutorial is also available.

### 2.3.1 Requirements

- **Operating system(s)**: tested on Windows, Linux and OSx operating systems
- **Programming language**: Java
- **Other requirements**: Java Runtime Environment 5.0 or higher

## Results and Discussion

We applied the GIANT plugin to two different scenarios of biological relevance: the contact network of hemoglobin structure and the co-expression network of *Vitis Vinifera*. These two different networks allow to study the relation between topology and function at two different levels of biological organization: structural and gene expression regulation level.

### 3.1 Scenario 1: Hemoglobin structure

Hemoglobin occupies a unique niche in the proteome: this multimeric protein drives the delicate balance of oxygen and carbon monoxide exchanges between blood and tissues. Hemoglobin, at odds with other molecular systems, essentially works by alone with any relevant interaction with other biomolecular systems. This implies we can fully trace back its functional properties from its structure, as witnessed by the large amount of literature dealing with the link between hemoglobin mutations and functional properties [19].

**Figure 3:**
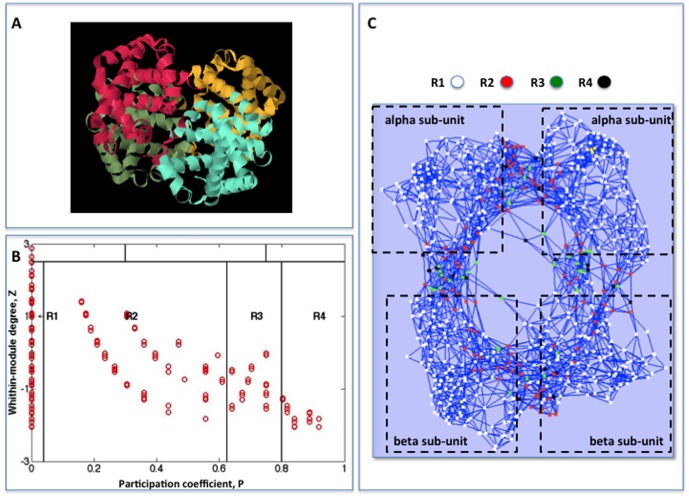
Guimerà-Amaral cartography for hemoglobin. A) X-ray-resolved molecular structure of hemoglobin protein (source Protein Data Bank PDB). B) Guimerà-Amaral cartography for hemoglobin; clusters are computed by spectral clustering algorithm. C) Hemoglobin protein contact network. The nodes colors correspond to their Guimerà-Amaral role.

Human hemoglobin structure (PDB code 1HBB) is made up of four sub-units (see Figure 3A), whose mutual spatial relations are at the basis of the so-called allosteric effect, due to the shift between two different configurations (T and R) at the basis of the cooperative binding and release of oxygen and carbon dioxide [20]. The identification of a peculiar topological role of the residues that connect the four sub-units can be considered a strong proof of the relevance of the GIANT approach.

Figure 2 reports the GIANT output for the analysis of the hemoglobin contact network. We transformed the protein structure data (spatial position of atoms) into a contact networks by putting an edge between residues (the nodes of the network) whose spatial distance of the corresponding 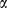 - carbons is comprised within 4 and 8 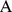, thus including only non-covalent intramolecular interactions [9], [12], [21].

Spectral clustering parts the contact network into four clusters, roughly corresponding to protein sub-units. Relying on this partition GIANT builds the modified Guimerà-Amaral cartography (see Figure 3B) and gives a pictorial representation of the network (see Figure 3C). Node colors correspond to their role in the modified Guimerà-Amaral cartography. R4 nodes (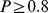, black colored) correspond to residues placed at the boundaries of the hemoglobin sub-units. White nodes correspond to region R1 (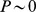, ultra-peripheral nodes). These nodes communicate only within their own module, remaining confined within the core of the hemoglobin sub-units. The unbiased identification of connector nodes by the algorithm is a proof-of-concept of the relation between structure topology and protein function. Spectral clustering of protein contact network produces characteristic 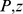 diagrams, (“dentist's chair”, due to their shape [9]); this shape has been tested to be strongly invariant across a 1420 protein molecules dataset [9]: the invariance of the protein cartography suggests the chance to extend the observed topology-function relation of hemoglobin to other protein systems. 4A). Notice that the quasi-parallel lines of the graph do not correspond to clusters and still defy a simple explanation.

### 3.2 Scenario 2: Gene co-expression network in *Vitis vinifera*

Fruit ripening processes involve an highly coordinated set of events at both macroscopic and molecular levels. In order to check the crucial steps in ripening, the genome-wide gene co-expression could give some important hints. The shift between different development patterns is mediated by specific genes, namely transcription factors [22], able to activate (inactivate) different development modules (clusters of co-expression genes).

We make the hypothesis that a similar model applies to *Vitis vinifera* ripening: genes co-expressed across different modules could be responsible for the activation of different plant development stages.

The gene expression dataset used to build the co-expression network comes from the Gene Expression Omnibus under the series entry GSE36128 [23]. It consists of 29550 genes of *Vitis vinifera* whose expression value has been measured using microarrays from 54 samples taken from different tissues and stages. Two samples refer to leaves senescence and to pollen, while the other 52 samples can be divided into two groups: 25 samples of red/mature/woody organs and 27 samples of green/vegetative organs.

In the plant co-expression network, a link occurs if the absolute value of the correlation between the gene expression profiles is greater than 0.8; this threshold minimizes the number of connected components for both green and red tissues.

**Figure 4:**
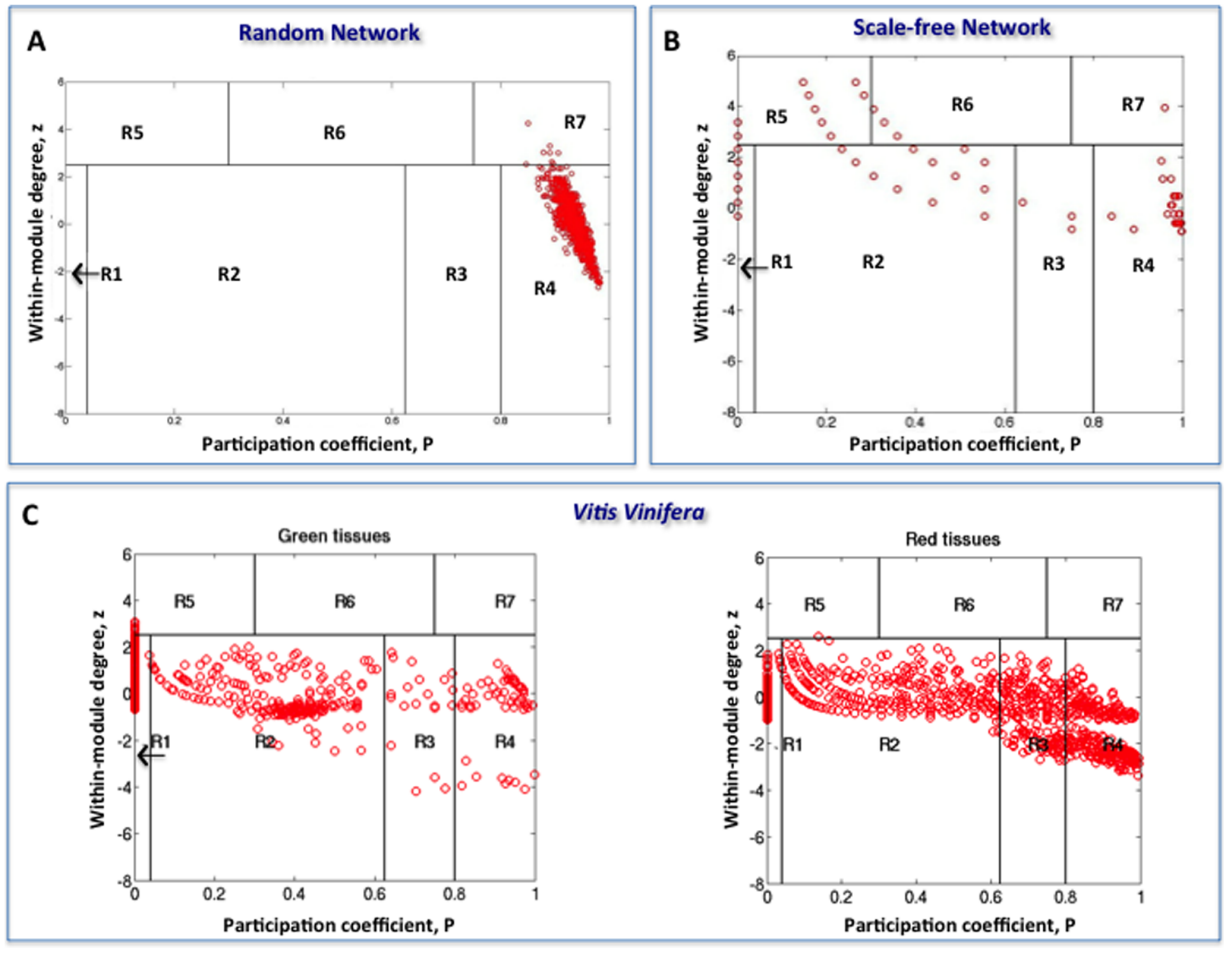
Guimerà-Amaral cartography. Guimerà-Amaral cartography for different networks: A) the random network of Erdos-Renyi with 1000 nodes and 50.000 edges [24]; B) the scale-free network of Barabàsi [25] with 1000 nodes and two clusters; C) the *Vitis Vinifera* co-expression network for vegetative (left) and woody organs (right).

We built two co-expression networks, one for the green tissues and one for the red tissues (see Figure 4C): the cartography of these networks resembles the ‘dentist's chair’ we described above for hemoglobin contact network. To test the nature of the shape invariance of the 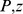 plane, we compared the co-expression networks with two simulated architectures corresponding to a random (Erdos-Renyi [24] Figure 4A) and a scale-free (Baràbasi) network with 1000 nodes and two clusters [25] (see Figure 4B).

In both scale-free Barabàsi and the *Vitis Vinifera* co-expression networks, we found again the characteristic dentist's chair in the 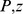 plane but not for random network. The strong invariance of the 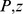 portraits is extremely intriguing, given it suggests the existence of still hidden mesoscopic principles of scale-free networks.

The R4 region of co-expression network for red tissues (ripened fruit) is enriched in transcription factors for the post-harvest development stage. Given the green tissues do not partecipate into fruit ripening and are made up of only one developmental stage, they do not show any relevant enrichment in transcription factors.

## Conclusions

It is noteworthy that a purely topological description of nodes, by the agency of the intermediate mesoscopic layer through modules identification, allows for the elucidation of the functional role of the biological network elements. This is particularly evident in the case of hemoglobin molecule where the network description exactly matched the structural role of the corresponding amino acid residues in terms of between- within- subunits location.

In the case of co-expression network, the topology function relationship is still hypothetical but suggests importante lines of experimentation The Cytoscape plugin GIANT provides a powerful tool for an accurate analysis of complex networks, offering a multiscale perspective from nodes local properties to general network architecture. This integration was possibile thanks to the quantitative description of the network at a mesoscopic (clustering) level, allowing a prompt view of nodes role. The plugin interface is simple and user-friendly and a practical video tutorials can be downloaded from the web site http://www.iasi.cnr.it/~dsantoni/GIANT/giant.html. Moreover, Cytoscape community accepted GIANT plugin, which is actually available for the download on the official Cytoscape APP Store. The modular architecture of the plugin allows to expand the system so to include other clustering algorithms.
